# Human Cytomegalovirus US28 Ligand Binding Activity Is Required for Latency in CD34^+^ Hematopoietic Progenitor Cells and Humanized NSG Mice

**DOI:** 10.1128/mBio.01889-19

**Published:** 2019-08-20

**Authors:** Lindsey B. Crawford, Patrizia Caposio, Craig Kreklywich, Andrew H. Pham, Meaghan H. Hancock, Taylor A. Jones, Patricia P. Smith, Andrew D. Yurochko, Jay A. Nelson, Daniel N. Streblow

**Affiliations:** a Vaccine and Gene Therapy Institute, Oregon Health and Science University, Beaverton, Oregon, USA; b Department of Microbiology and Immunology, Louisiana State University at Shreveport, Shreveport, Louisiana, USA; Princeton University; UNC-Chapel Hill; SUNY Upstate Medical University

**Keywords:** US28, hematopoiesis, human cytomegalovirus, latency, reactivation

## Abstract

Human cytomegalovirus (HCMV) infection of CD34^+^ hematopoietic progenitor cells (CD34^+^ HPCs) provides a critical reservoir of virus in stem cell transplant patients, and viral reactivation remains a significant cause of morbidity and mortality. The HCMV chemokine receptor US28 is implicated in the regulation of viral latency and reactivation. To explore the role of US28 signaling in latency and reactivation, we analyzed protein tyrosine kinase signaling in CD34^+^ HPCs expressing US28. US28-ligand signaling in CD34^+^ HPCs induced changes in key regulators of cellular activation and differentiation. *In vitro* latency and reactivation assays utilizing CD34^+^ HPCs indicated that US28 was required for viral reactivation but not latency establishment or maintenance. Similarly, humanized NSG mice (huNSG) infected with TB40E-GFP-US28stop failed to reactivate upon treatment with granulocyte-colony-stimulating factor, but viral genome levels were maintained. Interestingly, HCMV-mediated changes in hematopoiesis during latency *in vivo* and *in vitro* was also dependent upon US28, as US28 directly promoted differentiation toward the myeloid lineage. To determine whether US28 constitutive activity and/or ligand-binding activity were required for latency and reactivation, we infected both huNSG mice and CD34^+^ HPCs *in vitro* with HCMV TB40E-GFP containing the US28-R129A mutation (no CA) or Y16F mutation (no ligand binding). TB40E-GFP-US28-R129A was maintained during latency and exhibited normal reactivation kinetics. In contrast, TB40E-GFP-US28-Y16F exhibited high levels of viral genome during latency and reactivation, indicating that the virus did not establish latency. These data indicate that US28 is necessary for viral reactivation and ligand binding activity is required for viral latency, highlighting the complex role of US28 during HCMV latency and reactivation.

## Abstract (precis)

Human cytomegalovirus (HCMV) can establish latency following infection of CD34^+^ hematopoietic progenitor cells (HPCs), and reactivation from latency is a significant cause of viral disease and accelerated graft failure in bone marrow and solid-organ transplant patients. The precise molecular mechanisms of HCMV infection in HPCs are not well defined; however, select viral gene products are known to regulate aspects of latency and reactivation. The HCMV-encoded chemokine receptor US28, which binds multiple CC chemokines as well as CX_3_CR1, is expressed both during latent and lytic phases of the virus life cycle and plays a role in latency and reactivation. However, the specific timing of US28 expression and the role of ligand binding in these processes are not well defined. In this report, we determined that US28 is required for reactivation but not for maintaining latency. However, when present during latency, US28 ligand binding activity is critical to maintaining the virus in a quiescent state. We attribute the regulation of both latency and reactivation to the role of US28 in promoting myeloid lineage cell differentiation. These data highlight the dynamic and multifunctional nature of US28 during HCMV latency and reactivation.

## INTRODUCTION

Human cytomegalovirus (HCMV) is a betaherpesvirus that infects 44 to 100% of the population (1). Typically, primary infection results in asymptomatic disease. Following acute infection, HCMV persists in hematopoietic progenitor cells (HPCs), endothelial cells, and myeloid lineage cells for the lifetime of the infected individual. Latent HCMV can reactivate under specific conditions of immunosuppression, especially in patients undergoing immunosuppressive therapy following transplantation. As such, HCMV remains a significant cause of morbidity and mortality in solid-organ (SOT) and allogeneic hematopoietic stem cell (HSCT) transplant recipients (2, 3). Infection in HSCT patients is often associated with myelosuppression and graft failure due to a number of complicating factors that involve bone marrow hypoplasia requiring treatment to prevent anemia and thrombocytopenia. Leukopenia caused by HCMV reactivation puts many patients at risk of secondary life-threatening infections (4). Predicting HCMV reactivation and disease, including myelosuppression, in patients is difficult, as virus-associated and host-specific mechanisms remain unclear. In addition, to date no FDA-approved vaccine exists against HCMV, and treatment for reactivation and disease in HSCT recipients is limited to antivirals such as valganciclovir and others that have potent toxic side effects, including myelosuppression. Therefore, in order to develop novel therapies to prevent HCMV disease in transplant patients, there is a significant need to identify the viral mechanisms involved in HCMV reactivation and myelosuppression.

Following HCMV infection of CD34^+^ HPCs, the virus is maintained in a latent state that can be reactivated to replicate in myeloid-lineage cells. The mechanisms by which these events occur are currently only partially described. A limited number of HCMV gene products have been categorized as latency-associated gene products, including virus interleukin-10 (vIL-10) (5), LUNA (6, 7), UL133/138 (8–11), a subset of viral miRNAs (12, 13), and the chemokine receptor US28 (14, 15). A recent transcriptome sequencing (RNA-seq) approach identified detectable expression of 41 HCMV genes with high confidence at 2 days postinfection of CD34^+^ HPCs (16). Expression of many of these genes was lower at 6 days postinfection, which is consistent with previous data that demonstrated that infection of CD34^+^ HPCs induces a brief stage of viral gene expression after which most of the viral genome is silenced, probably through genome modification. Reactivation of HCMV from latency is associated with derepression of the viral genome (17–19) that results in activation of the viral immediate-early genes through alternative promoter sequences (20).

Chemokines and their receptors mediate cellular signaling, immune cell migration in response to inflammation, and multiple stages of hematopoiesis. HCMV disrupts the cellular chemokine/receptor axis by encoding viral chemokines/receptor homologues as well as by inducing host chemokines and regulating host chemokine receptor expression. The viral protein US28 uniquely binds both CC-chemokines (i.e., RANTES) and the CX_3_CL1 chemokine Fractalkine (21–24) and directs cellular pathways using both ligand-independent and ligand-dependent signaling. Ligand-dependent signaling, mediated through differential G-protein coupling, is also highly cell type specific and likely has distinct roles depending on the viral life cycle and cellular differentiation stage. Importantly, US28 is expressed in naturally infected human peripheral blood cells during periods of latency (25) and during reactivation in lung transplant recipients (26). US28 is also expressed in models of HCMV latency using CD34^+^ HPCs and monocytes as well as in monocyte-derived macrophages during active infection scenarios (14, 27–30). Combined, these data indicate that US28 is expressed during latent and lytic replication and, as such, may play an important role in viral latency and reactivation. Previous reports have suggested that US28 is required for HCMV latency maintenance and that this activity is dependent upon constitutive signaling (14, 15). Here, we demonstrate that US28 is required for HCMV reactivation from latently infected CD34^+^ HPCs and latently infected humanized mice (huNSG). Viral genomes were maintained in both systems during latency, indicating that US28 is not required for latency establishment or maintenance. However, in the absence of US28 ligand-binding activity the virus replicated in CD34^+^ HPCs, suggesting that ligand binding suppresses virus replication to maintain latency. We also demonstrate that US28 expression alone is sufficient to drive CD34^+^ HPC differentiation toward a myeloid lineage *in vitro* and that loss of US28 during latent infection significantly blocked myeloid cell differentiation in huNSG mice. Our findings suggest that US28 can modulate viral latency and acts as a sensor to both alter the cellular differentiation state and promote viral reactivation.

## RESULTS

### US28 signals in both ligand-dependent and -independent manners in CD34^+^ HPCs.

**FIG 1:**
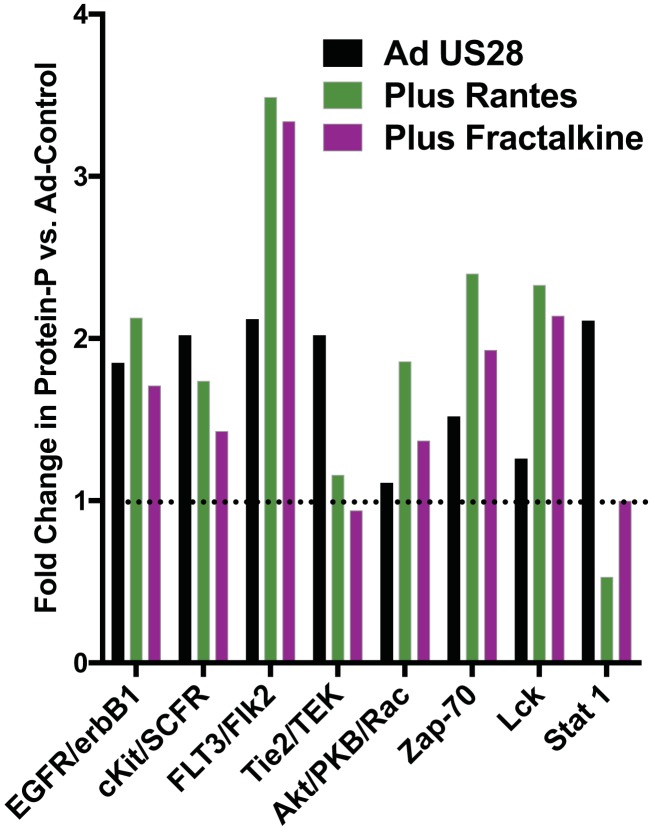
US28 promotes cellular kinase phosphorylation in CD34^+^ HPCs. CD34^+^ HPCs infected with Ad-US28 were left untreated or were treated with PBS, RANTES (CCL5), or Fractalkine (CX_3_CL1). HPCs infected with Ad-Empty that were treated with PBS were used as a background control for phosphorylation status. At 30 min posttreatment, cellular lysates were collected and analyzed using the PathScan RTK chip to quantify protein phosphorylation. The data are represented as fold change versus values for Ad-Empty and are representative of two independent experiments.

US28 signals through multiple pathways that influence cellular differentiation, including NF-κB, Src, FAK, Pyk2, RhoA, and Wnt (31–35). Previously, we uncovered that US28 signaling is ligand specific and that this phenomenon occurs in a cell type-specific manner through the promotion of differential G-protein coupling, which ultimately determines the nature of the signaling pathway activation (36). To understand the role that US28 plays in CD34^+^ HPCs, we sought to identify signaling pathways that are activated by US28 in this specific cell type and determine whether these pathways are activated by ligands. For this experiment, undifferentiated CD34^+^ HPCs were transduced with Ad-empty or Ad-US28. At 18 h postinfection, subsets of cells were either left untreated or incubated with 50 μg/ml RANTES (CCL5) or Fractalkine (CX_3_CL1) and harvested in lysis buffer at 30 min posttreatment. Cellular lysates were normalized for protein concentration and applied to PathScan RTK chips to quantify the phosphorylation of signaling proteins. Untreated Ad-empty infected cells were used as a background control to determine the fold change in protein phosphorylation for cells expressing US28 with or without ligands. US28 mediates an increase in phosphorylation status of several major cell regulatory pathway families, including EGFR/erbB1, c-Kit/SCFR, FLT3/FLK2, Tie2/TEK, Akt, Zap-70, Lck, and Stat1 (Fig. 1). Interestingly, these pathways are specifically regulated by ligand and ligand-independent mechanisms of US28. While phosphorylation of EGFR and c-Kit was induced by US28, this induction was not affected by the addition of US28 ligands. For FLT3, Akt, Zap-70, and Lck, both RANTES and Fractalkine increased their phosphorylation status, which is consistent with previous reports of ligand-dependent signaling by US28 through these and other related pathways. Interestingly, phosphorylation of Tie2 and Stat1 was induced by US28 in the absence of exogenously added ligands, and in fact, ligand addition reduced them to control levels or below. These data indicate that US28 signals in both ligand-dependent and independent manners in CD34^+^ HPCs, and these activities are specific based upon the mediated pathway. US28 ligand signaling most likely promotes cell survival pathways and reduces activation of signaling pathways, such as Stat1, that may promote differentiation. Thus, we hypothesize that US28 ligand signaling is important for maintaining the cells during latency.

### US28 is required for HCMV reactivation but not establishment and maintenance of the viral genome in CD34^+^ HPCs.

**FIG 2:**
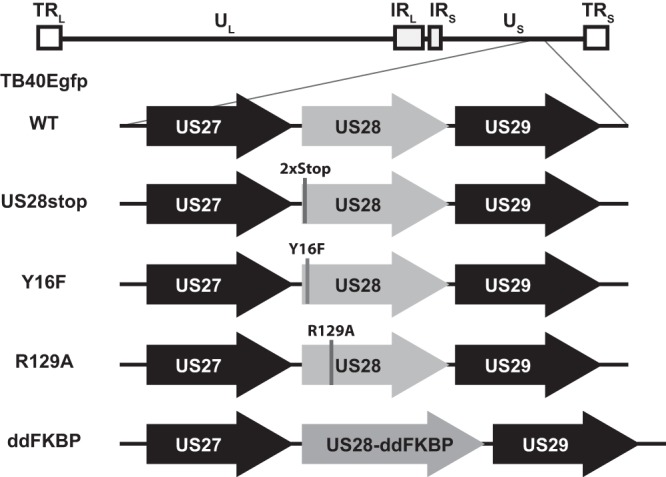
HCMV-TB40E-GFP constructs. A bacterial artificial chromosome containing the HCMV TB40E-GFP genome was used as the genetic backbone for recombineering of US28 mutants using the 2-step *galK-Kan* method. US28 mutations and ddFKBP C-terminal fusion are depicted.

**FIG 3:**
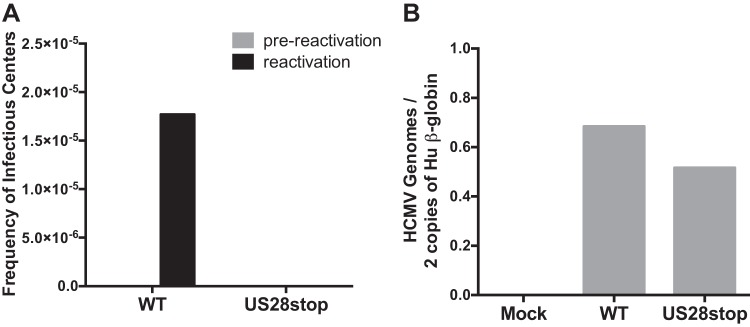
HCMV US28 is required for viral reactivation. CD34^+^ HPCs were infected with HCMV TB40E-GFP-WT or -US28stop at an MOI of 3. At 2 days postinfection (dpi), the cells were sorted by FACS for viable GFP^+^ CD34^+^ HPCs. HPCs were cultured for an additional 12 days in transwells over stromal cells (14 dpi) to establish latency. (A) Equal numbers of latently infected HPCs were either directly cocultured with NHDFs in cytokine-enriched media to induce viral reactivation (reactivation) or lysed and plated onto NHDFs (prereactivation) to assess the amount of virus present prior to reactivation. At 14 days postplating, the number of GFP-positive wells was determined by fluorescence microscopy, and the frequency of infectious centers was determined by ELDA software. (B) Total genomic DNA from latent HPCs was isolated, and quantitative real-time PCR was used to quantify the ratio of viral genomes (copies of HCMV UL141) to cellular genomes (per two copies of human [Hu] β-globin). Data are representative of three independent experiments; additional experiments are shown in Fig. S1.

To determine the role of HCMV US28 in latency and reactivation, we constructed a panel of recombinant HCMV strain TB40E-GFP viruses to disrupt US28 protein expression. We first blocked US28 expression by replacing two amino acid residues at the N terminus of the protein with in-frame contiguous stop codons or by inserting the FKBP destabilization domain (ddFKBP) at the C terminus (Fig. 2). To determine if US28 is required for either the establishment or maintenance of latency in CD34^+^ HPCs or is required for successful reactivation of the virus in progenitor cells, we infected CD34^+^ HPCs with HCMV TB40E-GFP or HCMV TB40E-GFP-US28stop and isolated green fluorescent protein-positive (GFP^+^) cells by fluorescence-activated cell sorting (FACS) at 2 days postinfection (dpi). CD34^+^ GFP^+^ HPCs were seeded into long-term bone marrow cultures in transwells over a layer of stromal feeder cells that supports HCMV latency (37). At 14 dpi, a limiting-dilution assay was used to determine the frequency of cells producing infectious virus during latency (prereactivation) compared to reactivation following coculture with fibroblasts in the presence of a cytokine cocktail that promotes myeloid cell differentiation. The number of GFP^+^ wells was counted weekly postcoculture and the frequency of reactivation calculated (37). In this culture system, CD34^+^ HPCs infected with HCMV TB40E-GFP-US28stop failed to reactivate, unlike wild-type (WT) virus (Fig. 3A; see also Fig. S1A and C in the supplemental material). Since a failure to reactivate can be caused by several mechanisms, we further explored whether this defect was due to the establishment of latency, viral genome maintenance, or defects during the reactivation program. To determine whether the viral genome is maintained in the absence of US28, total viral genomes were quantified by quantitative PCR (qPCR) in both WT- and ΔUS28-infected HPC populations at 14 dpi following latency culture and immediately prior to reactivation. As shown in Fig. 3B (as well as Fig. S1B and D), similar levels of viral genomes were present in the CD34^+^ HPCs at 14 dpi, indicating that US28 was not required for establishment of latency or the maintenance of latent genomes. This finding is in contrast to recent work using a US28 gene substitution virus that demonstrated a role for US28 only in latency maintenance (14).

10.1128/mBio.01889-19.1 FIG S1 HCMV US28 is required for viral reactivation. CD34^+^ HPCs were infected with HCMV TB40E-GFP-WT or -US28stop at an MOI of 3. At 2 days postinfection (dpi), the cells were sorted by FACS for viable GFP^+^ CD34^+^ HPCs. HPCs were cultured for an additional 12 days in transwells over stromal cells (14 dpi) to establish latency. (A and C) Equal numbers of latently infected HPCs were either directly cocultured with NHDFs in cytokine-enriched media to induce viral reactivation (reactivation) or lysed and plated onto NHDFs (prereactivation) to assess the amount of virus present prior to reactivation. At 14 days postplating, the number of GFP-positive wells was determined by fluorescence microscopy, and the frequency of infectious centers was determined by ELDA software. (B and D) Total genomic DNA from latent HPCs was isolated, and quantitative real-time PCR was used to quantify the ratio of viral genomes (copies of HCMV UL141) to cellular genomes (per two copies of hu β-globin). Download FIG S1, EPS file, 1.2 MB. Copyright © 2019 Crawford et al. 2019 Crawford et al. This content is distributed under the terms of the Creative Commons Attribution 4.0 International license.

10.1128/mBio.01889-19.2 FIG S2 US28 induces myeloid colony formation in CD34^+^ HPCs. (A and B) CD34^+^ HPCs were mock infected or infected with HCMV TB40E-GFP-WT or TB40E-GFP-ΔUS28 for 2 days. FACS-isolated viable GFP^+^ CD34^+^ HPCs were plated in Methocult H4434 at 500 cells/well and counted at 7 days. Data shown are average numbers of myeloid colonies per well for triplicate wells. (C) CD34^+^ HPCs were infected with Ad-US28 or Ad-Empty (control). At 24 hpi, cells were plated in Methocult H4434 for 7 days. Error bars represent standard deviations between three replicate wells per experiment. *P* values were determined by one-way ANOVA (A and B) or by *t* test (C) and are listed as exact values. Download FIG S2, EPS file, 1.2 MB. Copyright © 2019 Crawford et al. 2019 Crawford et al. This content is distributed under the terms of the Creative Commons Attribution 4.0 International license.

**FIG 4:**
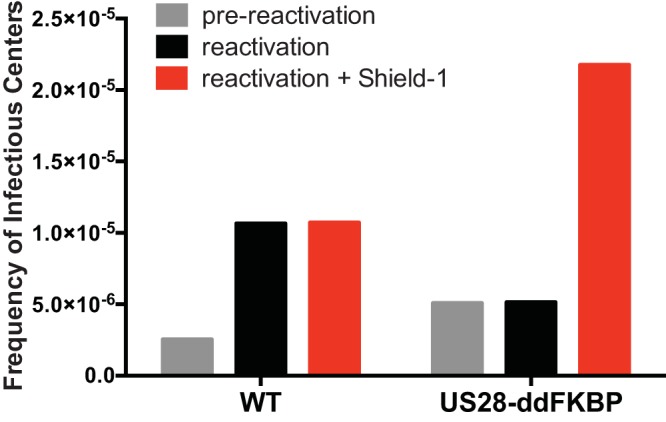
FKBP protein destabilization domain validates that HCMV US28 is required for viral reactivation. CD34^+^ HPCs were infected with HCMV TB40E-GFP-WT or -US28-ddFKBP and cultured to establish latency as described for Fig. 3. Following the establishment of latency, equal numbers of cells were either cocultured with NHDFs with or without 1 μM Shield-1 (reactivation conditions) or lysed and plated onto NHDFs (prereactivation). At 14 days postplating, the number of GFP-positive wells was determined by fluorescence microscopy, and the frequency of infectious centers was determined by ELDA software.

Therefore, to confirm that US28 was required specifically at the time of reactivation and not for latency maintenance, we constructed an inducible US28 mutant virus. By tagging US28 with FKBP (a small protein destabilization domain) (38), US28 expression can be specifically controlled by the addition and removal of the small molecule Shield-1. CD34^+^ HPCs were infected with either WT TB40E-GFP or TB40E-GFP-US28ddFKBP. In the absence of Shield-1 (lacking US28 expression), TB40E-GFP-US28ddFKBP cannot reactivate *in vitro*, which parallels HCMV deleted for US28. When US28 expression was restored at the time of reactivation by the addition of Shield-1, the virus was able to reactivate normally, in stark contrast to the lack of reactivation in the absence of the stabilizing compound (Fig. 4). These data further demonstrate that US28 is required for reactivation from latency rather than latency maintenance.

### US28 ligand binding activity promotes viral latency.

**FIG 5:**
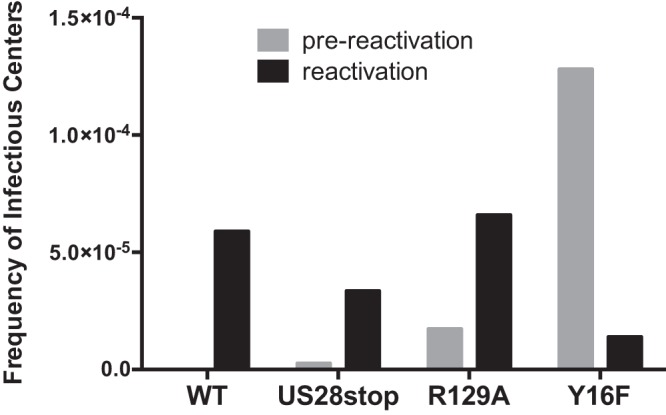
US28 ligand binding is required for maintenance of latency. CD34^+^ HPCs were infected with HCMV TB40E-GFP-WT, -US28stop, US28-R129A, or -US28-Y16F and cultured to establish latency as described for Fig. 3. Following the establishment of latency, equal numbers of cells were either cocultured with NHDFs in cytokine-enriched media (reactivation conditions) or lysed and plated onto NHDFs (prereactivation). At 21 days postplating, the number of GFP-positive wells was determined by fluorescence microscopy, and the frequency of infectious centers was determined by ELDA software.

Since US28 has both ligand-independent and ligand-dependent signaling and can specifically bind chemokines to direct cellular signaling pathways, we explored whether ligand binding activity or constitutive signaling are important for HCMV reactivation from latency. Two US28 signaling mutants were generated on the WT HCMV TB40E-GFP backbone to assess the role of US28 ligand-dependent binding (Y16F) versus constitutive signaling (R129A) (Fig. 2). Infection of CD34^+^ HPCs with TB40E-GFP-US28-R129A results in prereactivation and reactivation levels of virus comparable to those observed for WT-infected HPCs (Fig. 5), suggesting that a lack of US28 constitutive signaling does not alter the ability of the virus to establish and maintain latency or reactivate, which contrasts with recent findings using a monocyte/THP-1 system (15). However, when HPCs were infected with TB40E-GFP-US28-Y16F, which lacks US28 ligand binding activity, higher levels of infectious virus were detected during latency culture, suggesting an ongoing lytic infection for this viral mutant (Fig. 5, prereactivation). This finding indicates that ligand binding of US28 is required to establish or maintain the latent state. The latency phenotype of this altered signaling mutant, therefore, is in stark contrast to the failure to reactivate from latency when US28 is completely disrupted. These data demonstrate that US28 has a complex and dynamic role in HCMV latency and reactivation.

### US28 is necessary for HCMV reactivation *in vivo* in huNSG mice, and ligand binding is required to maintain latency.

**FIG 6:**
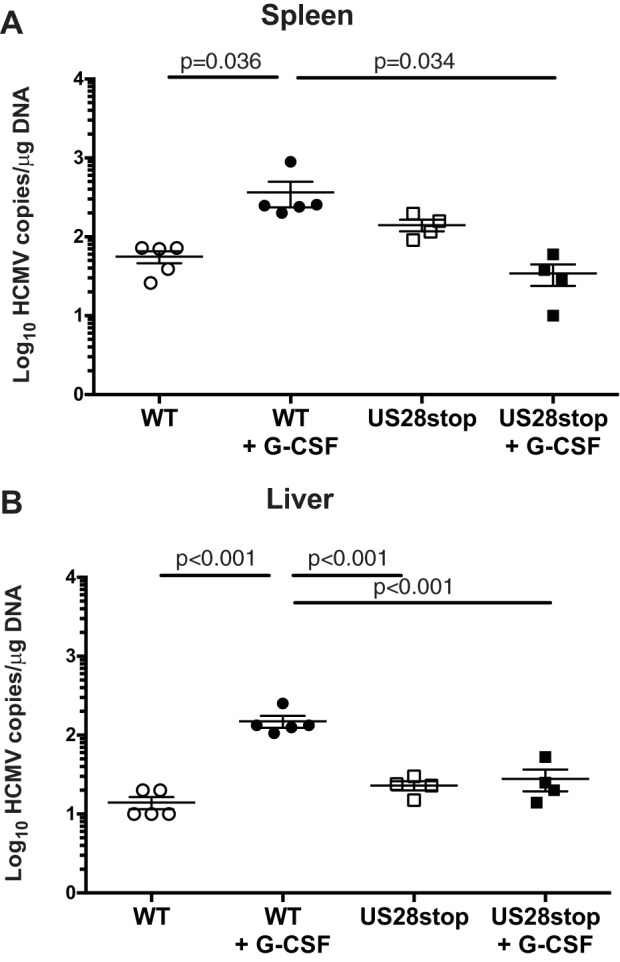
HCMV US28 is required for reactivation *in vivo.* Humanized NSG mice were injected with fibroblasts infected with either HCMV TB40E-GFP-WT or -US28stop (*n* = 8 to 10 per group). At 8 weeks postinfection, half of the mice were treated with G-CSF and AMD-3100 to induce cellular mobilization and promote HCMV reactivation. Control mice were left untreated. At 1 week postmobilization, mice were euthanized and tissues were collected. Total DNA was extracted using DNAzol, and HCMV viral load was determined by qPCR on 1 μg of total DNA prepared from spleen (A) or liver (B) tissue. Error bars represent standard deviations between average DNA copies from two (A) or four (B) tissue sections for individual animals. All samples were compared by one-way analysis of variance (ANOVA) within experimental groups (nonmobilized versus mobilized [+G-CSF] for each virus and between all virus groups for both nonmobilized and mobilized conditions). *P* values are listed for significant comparisons where *P* < 0.05.

**FIG 7:**
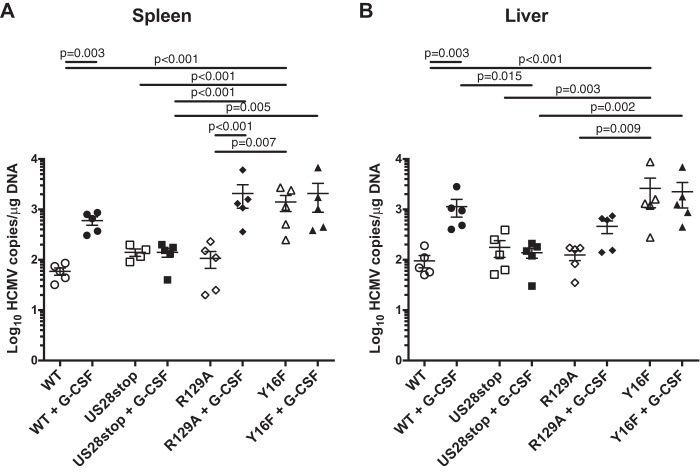
HCMV US28 ligand binding is required to maintain latency *in vivo.* Humanized NSG mice were injected with fibroblasts infected with either HCMV TB40E-GFP-WT, -US28stop, -US28-R129A, or -US28-Y16F (*n* = 10 per group). At 8 weeks postinfection, half of the mice were treated with G-CSF and AMD-3100 to induce cellular mobilization and promote HCMV reactivation. Control mice were left untreated. At 1 week postmobilization, mice were euthanized and tissues were collected. Total DNA was extracted using DNAzol, and HCMV viral load was determined by qPCR on 1 μg of total DNA prepared from spleen (A) or liver (B) tissue. Error bars represent standard deviations between average DNA copies from two (A) or four (B) tissue sections for individual animals. All samples were compared by one-way ANOVA within experimental groups (nonmobilized versus mobilized [+G-CSF] for each virus and between all virus groups for both nonmobilized and mobilized conditions). *P* values are listed for significant comparisons where *P* < 0.05.

To further explore the complex roles of US28 in latency and reactivation, we used the NOD-*scid* IL2Rγc null (huNSG) humanized mouse model developed by our group. huNSG mice are engrafted with human CD34^+^ HPCs and are a robust model of HCMV latency and reactivation (11, 39, 40). huNSG mice are infected by intraperitoneal injection of HCMV-infected fibroblasts and allowed 8 weeks to develop latent infection. To mimic natural reactivation conditions, half of the animals in each group are treated with granulocyte-colony-stimulating factor (G-CSF) and the CXCR4 inhibitor AMD3100 to mobilize bone marrow-derived cells, which promotes widespread tissue dissemination of HCMV-infected cells and stimulates differentiation and virus reactivation similarly to what has been observed in humans undergoing the same mobilization protocol (41). To determine whether US28 is required for reactivation *in vivo*, we infected huNSG mice with HCMV TB40E-GFP or HCMV TB40E-GFP-US28stop. At 8 weeks postinfection, half of the mice were mobilized with G-CSF and AMD3100, and the remainder were untreated. Spleen and liver tissue was harvested from both groups at 1 week after mobilization. Tissue HCMV viral loads were determined by real-time qPCR. Figure 6 demonstrates that HCMV genome levels were similar in HCMV TB40E-GFP- and HCMV TB40E-GFP-US28stop-infected nonmobilized mice, indicating that US28 is not required to establish or maintain latency in this model. However, following reactivation, viral genome levels were dramatically lower in both liver and spleen from the TB40E-GFP-US28stop-infected animals than those infected with the WT, indicating that US28 is required for reactivation. A separate cohort of huNSG mice, examined using CD34^+^ HPCs from an independent donor, verifies this finding (Fig. 7). HCMV DNA levels in humanized mice infected with TB40E-GFP-US28-R129A, a virus that lacks constitutive US28 signaling, was very similar to levels for the WT after reactivation (Fig. 7). However, TB40E-GFP-US28-Y16F showed higher prereactivation HCMV DNA levels than the WT or any other mutant, suggesting that the virus was unable to remain latent (Fig. 7). These findings parallel our findings in the CD34^+^ HPC *in vitro* latency/reactivation model (Fig. 3 and 5), demonstrating that US28 ligand binding activity is required to establish or maintain latency and that US28 expression is required for reactivation from latency.

### US28 promotes CD34^+^ HPC differentiation toward the myeloid lineage.

**FIG 8:**
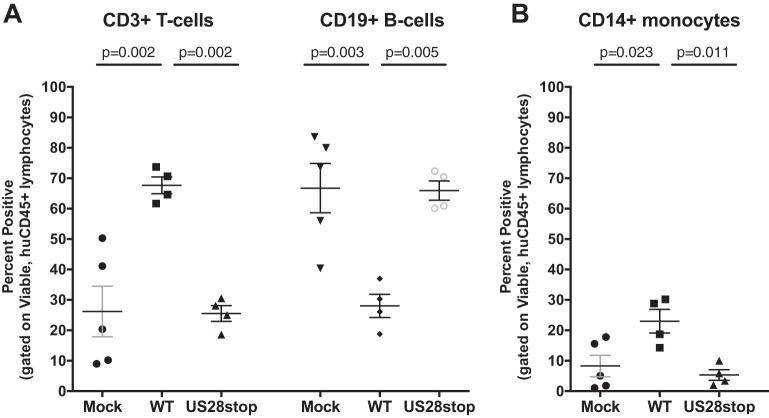
US28 alters hematopoiesis *in vivo* in huNSG mice. Humanized NSG mice were injected with mock-, HCMV TB40E-GFP-WT-, or HCMV TB40E-GFP-ΔUS28-infected fibroblasts (*n* = 4 to 5 per group). At 8 weeks postinfection, phenotypic analysis of human CD45^+^ blood leukocytes or splenocytes was performed by flow cytometry for T cell (CD3), B cell (CD19), and monocyte/macrophage (CD14) populations. Data from mock- and WT-infected huNSG mice shown here were previously published (39). Error bars represent standard deviations between individual animals. All samples were compared by one-way ANOVA between all virus groups, and *P* values are listed for significant comparisons where *P* < 0.05.

Based upon the above-described findings, we hypothesized that US28 signaling is required to push HCMV latently infected cells toward the myeloid differentiation pathway, which is conducive for effective viral reactivation. HCMV infection in HSCT patients is associated with myelosuppression, indicating that HCMV alters normal hematopoiesis through direct and/or indirect mechanisms. In order to determine whether US28 plays a role in myelopoiesis, we performed a phenotypic analysis of lymphocytes and myeloid-lineage cells from huNSG mice that were mock infected or latently infected with either HCMV TB40E-GFP or HCMV TB40E-GFP-US28stop. Humanized mouse blood and splenocytes were collected at 8 weeks postinfection (during clinical latency) and analyzed by multicolor flow cytometry. Human cells were demarked as viable, muCD45^−^, and huCD45^+^ and then further delineated with markers of B cell (CD19), T cell (CD3), and monocyte/macrophage (CD14) lineages. HCMV infection reduced the frequency of B cells while increasing the frequency of CD14^+^ monocytes within the total human CD45 leukocyte compartment (Fig. 8) but did not significantly affect the frequency of T cells. Interestingly, US28 drives each of these phenotypic shifts, as engrafted mice infected with HCMV-TB40E-ΔUS28 displayed a phenotype most closely resembling that of mock-infected mice. Importantly, these data demonstrate that, through US28, HCMV preferentially promotes a myeloid-lineage phenotype in a process that is critical for promoting viral reactivation from latency in HPCs.

**FIG 9:**
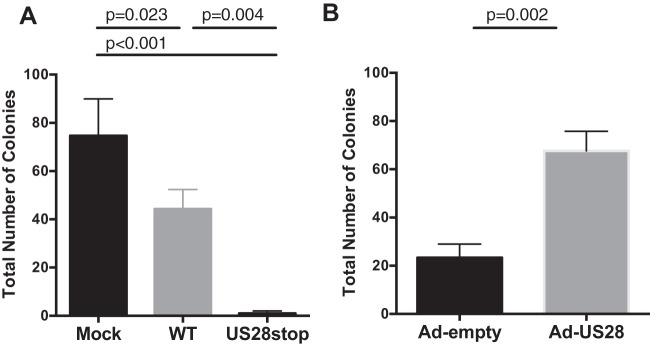
US28 induces myeloid colony formation in CD34^+^ HPCs. (A) CD34^+^ HPCs were mock infected or infected with HCMV TB40E-GFP-WT or TB40E-GFP-ΔUS28 for 2 days. FACS-isolated viable GFP^+^ CD34^+^ HPCs were plated in Methocult H4434 at 500 cells/well and counted at 7 days. Data shown are average numbers of myeloid colonies per well for triplicate wells. Data are representative of three independent experiments. (B) CD34^+^ HPCs were infected with Ad-US28 or Ad-Empty (control). At 24 hpi, cells were plated in Methocult H4434 for 7 days. Data are representative of two independent experiments. Error bars represent standard deviations between three replicate wells per experiment. *P* values were determined one-way ANOVA (A) or by *t* test (B) and are listed as exact values. Replicate experiments are shown in Fig. S2.

To validate that US28 is responsible for promoting myeloid lineage differentiation of HPCs directly, we utilized an *in vitro* CD34^+^ HPC myeloid colony formation assay. To determine whether US28 directly influences myelopoiesis, CD34^+^ HPCs were infected with HCMV TB40E-GFP or HCMV TB40E-GFP-US28stop for 48 h. FACS-purified viable CD34^+^ GFP^+^ HPCs were plated in Methocult H4434, and colonies were counted at 7 and 14 days. As previously described, HCMV infection substantially inhibits myeloid colony formation (42–44) (Fig. 9A and Fig. S2A and B), supporting a direct role for HCMV in myelosuppression. Infection with HCMV lacking US28 further suppresses colony formation, suggesting that US28 protects HCMV-infected HPCs, allowing them to undergo differentiation and/or to directly induce differentiation. To examine this issue, we performed a similar colony formation assay using adenoviral vectors to express US28 in HPCs. Colony formation was ∼3-fold higher in Ad-US28 cells than in the Ad-empty control, indicating that US28 can directly promote myeloid differentiation (Fig. 9B and Fig. S2C).

## DISCUSSION

The HCMV-encoded chemokine receptor US28 is expressed during latency and under conditions that favor reactivation and lytic viral replication. Here, we explored the role of US28 ligand binding activity in latency and reactivation using both *in vitro* and *in vivo* systems. Our results using a recombinant virus containing two contiguous stop codons in the US28 open reading frame indicate that US28 is required for HCMV reactivation. However, HCMV genome copy number was maintained during latency for the US28 mutant virus at levels comparable to those of the WT, suggesting that US28 is not necessary for the establishment or maintenance of latency. This finding was confirmed using a recombinant HCMV containing an in-frame destabilization domain (FKBP) that only reactivates in the presence of the compound Shield-1, which we used to stabilize US28 only during the reactivation process. However, the role for US28 during latency is complicated. A virus expressing US28 lacking constitutive activity (R129A) had no effect *in vitro* or *in vivo*, as the virus establishes and reactivates from latency similarly to the WT. However, in CD34^+^ HPCs infected *in vitro* or in humanized mice infected with a recombinant virus containing a mutation that blocks US28 ligand binding activity (Y16F), the virus failed to enter into a latent state and instead continuously replicates. This virus appears to take on a perpetual reactivation state similar to that described for viruses lacking UL138 (8, 11). The implications of how US28 ligand binding activity affect latency processes is discussed in more detail below. We also found that HCMV dramatically affects hematopoiesis in humanized mice. We show here that HCMV drives hematopoietic differentiation *in vivo* toward CD14^+^ monocytes and that this effect was mitigated in mice infected with a recombinant HCMV lacking expression of US28. Interestingly, this effect is significant in the background of low basal levels of circulating monocyte engraftment found in the huNSG model. Additional studies to assess the effect of specific monocyte subsets are being explored using humanized mouse models with enhanced monocyte reconstitution. A similar finding was observed using an *in vitro* colony formation assay, where HCMV infection reduces myeloid colony formation relative to that of uninfected cells, and this effect was dramatically enhanced in the absence of US28. CD34^+^ HPCs transduced with an adenoviral vector expressing US28 promoted myeloid colony formation, which supports a direct role for US28 in this process. Thus, we demonstrate here that US28 is required for HCMV reactivation, that maintenance of latency is ligand dependent, and that US28 promotes hematopoiesis in CD34^+^ HPCs. Thus, US28 most likely promotes viral latency and drives the differentiation of these cells toward a reactivation-competent phenotype.

US28 has been proposed to have a number of complex functions in infected cells that undoubtedly contribute to the biology of CMV infection, including general cellular activation, chemotactic migration, chemokine scavenging, cellular adherence, viral latency/reactivation, and tumor promotion (45–47). Both constitutive and ligand-dependent signaling have been detected for US28, and these different signaling mechanisms lead to unique functional outcomes. For instance, US28-induced cellular migration occurs in a ligand-dependent manner and is not impacted by the R129A mutation (47). Interestingly, the ligand required for this migration event is cell type specific; US28-expressing smooth muscle cells migrate in response to CC chemokines and are inhibited by the CX_3_C chemokine Fractalkine, but the opposite is true for macrophages (31). Interestingly, we did not observe differences in US28 signaling through these different chemokine family members in CD34^+^ HPCs (Fig. 1). The role of US28 in latency and reactivation has been investigated using several different HCMV latency systems. In primary monocytes or the monocytic cell line THP-1, deletion of US28 was associated with uncontrolled immediate-early gene expression in a reactivation-like phenotype (15). This reactivation phenotype for the deleted virus was prevented through complementation with WT or Y16F US28 provided in *trans* but not with US28 R129A, which lacks constitutive activity. These findings in monocytes are not consistent with our findings in CD34^+^ HPCs or humanized NSG mice, where the R129A mutant had a phenotype similar to that of WT virus. In Kasumi-3 and CD34^+^ cells, deletion of US28 was also linked to a higher background level of reactivation (14). These discrepancies likely represent either differentiation state disparities between the different cell systems or unforeseen issues with deletion of the entire US28 gene sequence, since US28 is encoded on a polycistronic transcript that also includes US27 and US29. Thus, it is possible that deletion of US28 sequences alter expression of these other genes, but there are no antibodies currently available to assess possible effects on US27 and US29. Since we think that HCMV uses US28 as a sensor and modulator of the cellular differentiation state, the fact that we observe these differences between the latency systems is not unexpected. This is also highlighted by the fact that US28 signaling and function occur in a cell type-specific manner (31, 48).

Our results suggest that US28 ligand binding and signaling act to fine-tune HPC cellular differentiation toward a phenotype that promotes the capacity of the virus to reactivate, namely, toward a myeloid lineage, while also maintaining some degree of stemness. It is worth mentioning that other chemokines (CCL3, CCL15, and CXCL12) play important roles in hematopoiesis and myeloid cell differentiation (49–51). While it is unclear precisely why US28 ligand binding activity (and signaling) is required to maintain latency, US28 signals through multiple pathways that influence cellular differentiation, including NF-κB, Src, FAK, Pyk2, RhoA, and Wnt. Based upon our analysis, we propose the following model of how US28 and ligand binding influence latency and hematopoiesis. Reactivation from CD34^+^ HPCs requires that the cell be differentiated down the myeloid lineage toward monocyte/macrophages and/or dendritic cells. US28 signaling in CD34^+^ HPCs caused the phosphorylation of FLT3, which, depending upon the level of FLT3 activation, can promote cellular differentiation toward granulocyte-macrophage precursors (52, 53). During HCMV reactivation, expression of UL7 may also help to drive this phenotype (39). US28 ligand binding promoted higher levels of FLT3 phosphorylation than that without ligands (Fig. 1), which is consistent with data presented in Fig. 9 that demonstrates that US28 promotes myeloid colony formation. Dendritic cell maturation from myeloid-lineage progenitors requires Stat1 activation (54). Interestingly, US28, in the absence of ligands, was associated with an increase in Stat1 phosphorylation, which suggests that US28 signaling in the absence of ligand, and possibly constitutive activity, promotes the formation of a cell type that is capable of productive reactivation. This scenario would be supported by our finding that infection with a recombinant US28 lacking ligand binding (Y16F) was associated with a perpetual replication state *in vitro* (Fig. 5) and *in vivo* (Fig. 7). However, the effect of Stat1 phosphorylation was mitigated by US28 ligand binding, indicating that ligand binding activity promotes or maintains stemness of the latently infected cell, which, in turn, would promote viral quiescence. CD34^+^ HPCs are also capable of differentiating toward an endothelial cell lineage. Signaling through Tie2, the receptor for Angiotensin-1, has been shown to promote endothelial cell differentiation from stem cells (55, 56). US28 expression in CD34^+^ HPCs promotes Tie2 phosphorylation, but in the presence of either RANTES or Fractalkine this phosphorylation event was diminished to background levels. This also suggests that US28 ligand binding activity is steering the infected CD34^+^ cells away from becoming endothelial-like, which may not be a productive phenotype for either reactivation/replication, dissemination, or virus transmission. Related to this observation, we have previously demonstrated that Src-family kinases are activated by US28 signaling in a ligand-dependent manner (33). In the current report, US28 promoted the phosphorylation of Lck, a src-family kinase (SFK) present in HPCs. This activity was ligand dependent, as both Fractalkine and RANTES were capable of activating this kinase. Interestingly, epidermal growth factor receptor (EGFR) not only has been shown to be required for HCMV entry into CD34^+^ HPCs but also plays a key role in maintaining latency by blocking immediate-early gene synthesis (8, 57). We suggest that US28-mediated activation of SFKs coopts growth factor receptor signaling pathways, such as EGFR or ZAP70 (58), to prevent premature reactivation. Similarly, US28 activation of c-Kit would promote an HPC self-renewal phase and reduce the likelihood that the latently infected cell would initiate cell death pathways, since even slight changes in c-Kit can profoundly affect HPCs (59). Thus, we suggest that US28 ligand binding activity promotes viral quiescence in CD34^+^ HPCs by pushing them toward a myeloid lineage, therefore ensuring productive reactivation at the same time that the vGPCR is inducing a phenotype to promote viral quiescence prior to when the proper reactivation conditions are met. Normal hematopoiesis is a delicate balance between four cellular states, including self-renewal, quiescence, differentiation, and apoptosis. HCMV has developed the ability to navigate and manipulate these stages to utilize HPCs and downstream lineages as a viable means of persistence. Thus, determining the function of US28 signaling in each of these stages is ongoing and the subject of further investigation.

## MATERIALS AND METHODS

### Cells.

MRC-5 human fibroblasts and normal human dermal fibroblasts (NHDFs) were cultured in Dulbecco’s modified Eagle’s medium (DMEM) supplemented with 10% fetal bovine serum (FBS), penicillin, streptomycin, and glutamine and maintained at 37°C in 5% CO_2_. CD34^+^ hematopoietic progenitor cells (HPCs) were isolated from deidentified fetal liver obtained from Advances Bioscience Resources using CD34^+^ magnetic bead separation (Miltenyi) as previously described (60).

### Adenovirus vectors.

Adenovirus (AdV-E1A^−^/E3^−^) vectors expressing US28-HA were previously described (31). US28 expression from these vectors is driven by a Tet-responsive enhancer within a minimal CMV promoter. Recombinant adenoviruses were expanded on 293-Cre cells, and titers of the viral stocks were determined on 293 cells by limiting-dilution assay. Gene expression was driven by coinfection with an Ad-transactivator as previously described (31–34).

### HCMV recombinant virus construction.

The bacterial artificial chromosome (BAC) containing the HCMV strain TB40E-GFP was engineered to constitutively expressed green fluorescent protein under the simian virus 40 (SV40) promoter (11). Recombinant TB40E-GFP-US28stop, TB40E-GFP-US28-Y16F, TB40E-GFP-US28-R129A, and TB40E-GFP-US28-ddFKBP, shown in Fig. 2, were cloned using a two-step positive/negative selection BAC recombineering method to create the mutation (36). TB40E-GFP-US28stop contained 2 contiguous stop codons at the beginning of the US28 open reading frame. TB40E-GFP-US28-Y16F contains a mutation that prevents CC and CX3C chemokine ligand binding (31, 61), whereas TB40E-GFP-US28-R129A contains a mutation that abrogates constitutive signaling (62). TB40E-GFP-US28-ddFKBP contains an in-frame C-terminal fusion of US28 with the FKBP destabilization domain (ddFKBP) to allow temporal expression of US28 with the addition of Shield-1. In the first recombination step, recombinant bacteria (SW105) containing the TB40E-GFP BAC were transformed with a PCR product comprised of a cassette containing the galactokinase and kanamycin resistance genes (*galK-Kan^r^*) flanked by sequences homologous to the site of US28 insertion. Kanamycin resistance was used to select bacteria containing the primary insertion and verified by PCR of the US28 genomic region and sequencing to confirm the presence of the *galK-Kan^r^* cassette. In the second recombination step, the *galK-Kan^r^* cassette was replaced with a PCR product with sequence homology to the US28 recombination site containing the mutation or ddFKBP domain. The primers used to generate recombination products are listed in Table 1. Recombinants were selected on plates containing 2-deoxy-galactose, which is toxic in the presence of *galK*, and verified via PCR of the US28 genomic region and sequencing for the desired mutation. Virus was reconstituted from multiple individual recombinants by electroporation of purified BAC DNA into MRC-5 fibroblasts. Reconstituted virus was initially passaged and cloned by limiting dilution in 96-well plates using MRC-5 cells. Virus from at least two individual wells containing individual plaques was amplified in NHDFs and the US28 region sequenced to confirm the presence of the mutation. Virus stocks were prepared and titers determined as previously described (63).

**TABLE 1:** Primers and probe used in this study

Name	Orientation	Sequence
GalK-Kan insertion		
US28-2x Stop and US28- Y16F	Fwd	CGTGGACCAGGCGGTGTCCATGCACCGAGGGCAGAACTGGTGCTACC ATGCCTGTTGACAATTAATCATCGGCATAG
	Rev	GTCGTATTCAAACTCCGTCGTGAGTTCCGTG GTCGTCGTCGTCGGCGTCGGCGTCTCAGCAAAAGTTCGATTTA
US28-R129A	Fwd	CCGTGTACGTTACTCACTGCCTGTTTCTACGTGGCTATGTTTGCCAGT TTGTGTTTTATTACGGAGATTGCACTCCCTGTTGACAATTAATCATCG GCATAG
	Rev	GGCAAAGATCCACCAAAAAATACTGAAAAGGCAGGCCTGTTTTACAG GCCGATATCTCATGTAAACAATAGCGTACTCAGCAAAAGTTCGATTTA
US28-ddFKBP	Fwd	ACAGCATGAGCTTTTCGCGTCGGAGCTCGCCGAGCCGAAGAGAGAC GTCTTCCGACACGCTGTCCGACGAGGTGTGTCGCGTCTCACAAATTA TACCGCCTGTTGACAATTAATCATCGGCATAG
	Rev	TAAAAAAGCGCTACCTCGGCCTTTTCATACAAACCCCGTGTCCGCCC CTTTTTTCCCCGTGCCCGATATACACGATATTAAACCCACGACCATTT CCGTTCGATTCTCAGCAAAAGTTCGATTTA
Replacement		
US28-2x Stop	Fwd	CTCTTTCACGCGTCCGCCGCACA
	Rev	GAGCATTGAATCCGACGTCGC
US28-Y16F	Fwd	GGTGAACCGCTCATATAGACCA
	Rev	CAAGAAGTTGCCGACGGAAC
US28-R129A	Fwd	ACAACTCCCTAGCCAGCGTGCCGTGTACGTTACTCACTGCC
	Rev	TTGGTCACCACCATAAAGTGTGGAATGGCGATGATCAC
US28-ddFKBP	Fwd	TCGCCGAGCCGAAGAGAGACATCTTCCGACACGCTGTCCGACGAGG TGTGTCGCGTCTCACAAATTATACCGATGGGAGTGCAGGTGGAAACCA
	Rev	GGGCGGACACGGGGTTTGTATGAAAAGGCCGAGGTAGCGCTTTTTTA TTACTCAGCAAAAGTTCGATTTAGAATTCTTCCGGTTTTAGAAGCTCCA
HCMV detection		
UL141	Fwd	GATGTGGGCCGAGAATTATGA
	Rev	ATGGGCCAGGAGTGTGTCA
	Probe	FAM-CGAGGGAGAGCAAGTT-MGB

### Limiting-dilution reactivation assay.

Latency and reactivation was monitored in long-term cultures of CD34^+^ HPCs using methods previously detailed (37). Briefly, CD34^+^ HPCs were infected with HCMV TB40E-GFP or viruses containing mutations in US28 at a multiplicity of infection (MOI) equal to 3 for 48 h prior to isolation by fluorescence-activated cell sorting (FACS) using a FACSAria (BD FACS Aria equipped with 488-, 633-, and 405-nm lasers, running FACSDiva software) in order to obtain a pure population of viable GFP^+^ CD34^+^ HPCs. The cells were then cocultured in transwells above monolayers of irradiated M2-10B4, and S1/S1 stromal cells. At 14 days postinfection (dpi), HPCs were serially diluted in RPMI 1640 medium containing 20% FBS, 2 mM l-glutamine, 100 U/ml penicillin, 100 μg/ml streptomycin, 15 ng/ml granulocyte-colony-stimulating factor (G-CSF), and 15 ng/ml granulocyte-macrophage colony-stimulating factor (GM-CSF) and overlaid onto monolayers of NHDFs. To quantify the levels of prereactivation infectious virus, a fraction of the same HPC culture was mechanically disrupted and lysates added to NHDFs. For studies involving US28-ddFKBP, 1 μM Shield-1 (TaKaRa) was added to the reactivation medium and supplemented after 7 days. Cell cultures were microscopically visualized for the presence of GFP weekly, for up to 4 weeks, to assess the reactivation frequency from latently infected cells and the presence of preformed infectious virus by limiting-dilution assay (37).

### HCMV infection of humanized mice.

NOD-*scid* IL2Rγc null (NSG) mice (Jackson Laboratories) were bred in a specific-pathogen-free vivarium located at the Vaccine & Gene Therapy Institute at Oregon Health & Science University. All mouse procedures were performed according to an Institutional Animal Care and Use Committee approved protocol under the recommendations of the American Association for Accreditation of Laboratory Animal Care (AAALAC). Mice were housed in microisolator cages and fed sterile food and water. Mice were euthanized via CO_2_ administration according to AAALAC euthanasia guidelines. Humanized mice (huNSG) were generated by sublethal irradiation of 0- to 3-day-old neonates at 75 cGy using a ^137^Cs gamma irradiation source followed by intrahepatic injection of 10^5^ human CD34^+^ HPCs isolated from fetal liver as described previously (39, 60). Peripheral blood was drawn every 4 weeks, beginning at 8 weeks posttransplant, to assess the level of engraftment using flow cytometry. Engraftment efficiency is calculated as the percentage of human CD45^+^ cells out of total human and mouse CD34^+^ cells present in blood, and all experimental groups are normalized for human cell engraftment. Between 12 and 16 weeks postengraftment, mice were pretreated with 1 ml of 4% thioglycolate (Brewer’s medium; BD) by intraperitoneal (i.p.) injection. At 24 h posttreatment, mice were injected i.p. with the equivalent of two T150 flasks (i.e., 150-cm flasks) of TB40E-GFP wild-type- or mutant-infected NHDFs (a representative flask of virus was harvested at 24 h prior to injection to predetermine titers and match WT and mutant viruses). Experiment 1 (Fig. 6) used 1e6 PFU/mouse of each virus based on a 24-h 50% tissue culture infective dose (TCID_50_) titration. Experiment 2 (Fig. 7) used 1e5 PFU/mouse of each virus based on a 24-h TCID_50_ titration. At 8 weeks postinfection, half of the mice were treated with 100 μl of Neupogen (G-CSF; 300 mg/ml; Amgen) by subcutaneous pump and 125 μg of AMD3100 administered by i.p. injection in order to mobilize progenitor cells and promote HCMV reactivation (64). The other half of the mice in each group remained untreated to serve as comparators for viral levels during latency. At 1 week postmobilization, the mice were euthanized and bone marrow, blood, spleen, and liver were collected for further analysis.

### HPC colony formation assay.

Primary CD34^+^ HPCs were thawed and recovered overnight in stem cell medium (Iscove’s modified Dulbecco’s medium containing penicillin-streptomycin and stem cell cytokines [SCF, FLT3L, IL-3, and IL-6]) and infected with HCMV at an MOI of 3 or adenovirus vectors at an MOI of 1,000, as described above. At 48 hpi, viable GFP^+^ CD34^+^ HPCs were isolated by FACS and plated at 500 cells/ml in Methocult H4434 (Stem Cell Technologies) in 35-mm dishes in triplicate for myeloid colony assays. Total and specific myeloid colonies were enumerated manually at 7 and 14 days using a standard microscope. Error bars represent standard deviations for replicate wells. *P* values were determined by *t* test.

### Flow cytometry analysis.

Phenotypic analysis was performed on splenocytes or blood isolated from huNSG mice. At necropsy, spleen fractions were macerated and filtered through a 70-μm nylon cell strainer. Lymphocytes were isolated using Ficoll-Paque (GE Healthcare) and centrifuged at 600 × *g* (Beckman Centrifuge) for 15 min without braking. Lymphocytes were stained with Zombie Aqua viability dye (BioLegend), blocked in FACS buffer containing 5% each human and mouse serum, and stained with fluorescently labeled antibodies directed against human CD3 (UCHT1), CD14 (HCD14), CD19 (HIB19), and CD45 (HI30) and mouse CD45 (30-F11) (BioLegend). Samples were analyzed on an LSRII flow cytometer equipped with FACSDiva software (BD Biosciences), and data were analyzed using FlowJo software (Tree Star).

### Quantitative detection of HCMV viral DNA.

Total DNA was extracted from portions of mouse spleen or liver using DNAzol (ThermoFisher) as previously described (60) or from CD34^+^ HPCs using the two-step TRIzol (ThermoFisher) method by following the manufacturer’s protocol. Primers and probe recognizing HCMV *UL141* were used to quantify viral genomes by quantitative real-time PCR (primer and probe sequences are listed in Table 1) compared to a standard curve generated using purified HCMV BAC DNA. For tissue samples, 1 μg of total DNA was added to each reaction well of TaqMan FastAdvance PCR master mix (Applied Biosystems), and the samples were analyzed in triplicate on a StepOnePlus TaqMan PCR machine (Applied Biosystems) with an initial activation at 50°C for 2 min and 95°C for 20 s, followed by 40 cycles of 1 s at 95°C and 20 s at 60°C. TaqMan results were analyzed using ABI StepOne software and graphed using Prism 6 software. For HPC samples, the entire DNA fraction was analyzed in triplicate as described above, and data were normalized to total copies of human beta-globin as previously described (39).

### PathScan RTK arrays.

PathScan RTK signaling phosphoantibody array analysis (Cell Signaling) was performed according to the manufacturer’s protocol. Briefly, CD34^+^ HPCs were infected with adenoviruses expressing US28 and/or Tet transactivator as previously described for macrophages (31). At 18 hpi, cells were treated with RANTES (CCL5), Fractalkine (CX_3_CL1), or phosphate-buffered saline (PBS), and cellular lysates were collected at 30 min posttreatment. Protein lysate concentrations were normalized and incubated on the RTK antibody array chip for 2 h. The chip was washed, incubated with a detection antibody cocktail for 1 h, washed again, and incubated with a DyLight 680-linked streptavidin secondary antibody. Following washing and drying, chips were scanned using an Odyssey infrared imaging system (LI-COR) for spot intensity quantification using the Odyssey quantification software. Data are reported as fold change relative to the value for uninfected or untreated samples.
